# Supplementary material for: Arbitrary linear transformations for photons in the frequency synthetic dimension
Source: Nat Commun. 2021 Apr 23;12:2401. doi: 10.1038/s41467-021-22670-7 (PMC8065043; doi:10.1038/s41467-021-22670-7)
Supplement: Supplementary file 1 — Supplementary Information [file 41467_2021_22670_MOESM1_ESM.pdf]

Arbitrary linear transformations for photons in the frequency synthetic dimension

Siddharth Buddhiraju, Avik Dutt, Momchil Minkov, Ian A. D. Williamson, and Shanhui Fan
Ginzton Laboratory, Department of Electrical Engineering,
Stanford University, Stanford, California 94305, USA
(Dated: February 21, 2021)

SUPPLEMENTARY MATERIAL

Supplementary Note 1. Derivation of coupled-mode theory

Here, we derive the temporal coupled-mode theory formalism of Eqs. (1)-(3). We start with equation for the electric field $\mathbf{E}(\mathbf{r}, t)$ as derived from Maxwell's equations:

$$\nabla \times \nabla \times \mathbf{E}(\mathbf{r}, t) = \frac{1}{c^2}\frac{\partial^2}{\partial t^2}\left[(n(\mathbf{r})^2 + \delta\epsilon(\mathbf{r}, t))\mathbf{E}(\mathbf{r}, t)\right]. \tag{S1}$$

Here, $n(\mathbf{r})$ is the time-independent refractive index of the ring-waveguide structure and $\delta\epsilon(\mathbf{r}, t)$ is the time-dependent dielectric constant change induced by the electro-optic modulators in the form

$$\delta\epsilon(\mathbf{r}, t) = \sum_{l=1}^{N_\mathrm{f}} \delta\epsilon_l(\mathbf{r})\cos(l\Omega_\mathrm{R} t + \theta_l). \tag{S2}$$

The modes of the unmodulated ring are separated by the free spectral range (FSR) $\Omega_\mathrm{R} = c/n_\mathrm{g}R$, where R is the radius of the ring and n_g is the group index of the waveguide forming the ring. In the presence of weak modulation, the electric field can be expanded in the basis of the unmodulated modes:

$$\mathbf{E}(\mathbf{r}, t) = \sum_m a_m(t)\mathbf{E}_m(\mathbf{r})e^{i\omega_m t}, \tag{S3}$$

where $a_m(t)$ are the time-dependent complex amplitudes and $\mathbf{E}_m(\mathbf{r})$ are the modal profiles for the mth modes at frequencies $\omega_0 + m\Omega_\mathrm{R}$. Substituting Eq. (S3) into Eq. (S1), we obtain

$$\begin{aligned}\sum_m a_m(t)e^{i\omega_m t}\nabla \times \nabla \times \mathbf{E}_m = \frac{n^2}{c^2}\sum_m \mathbf{E}_m\left(\frac{\partial^2}{\partial t^2}a_m(t)e^{i\omega_m t}\right)\\ + \sum_{m,l}\frac{\delta\epsilon_l}{c^2}\mathbf{E}_m\left(\frac{\partial^2}{\partial t^2}a_m(t)e^{i\omega_m t}\cos(l\Omega_\mathrm{R} t + \theta_l)\right)\end{aligned} \tag{S4}$$

Using the fact that the unmodulated modes obey Maxwell's equations, i.e., $\nabla \times \nabla \times \mathbf{E}_m = -(n^2(\mathbf{r})\omega_m^2/c^2)\mathbf{E}_m$ as well as the slowly varying envelope approximation where we ignore terms involving the second derivative of a_m, we obtain

$$\sum_m e^{i\omega_m t}\left[2in^2\omega_m\mathbf{E}_m\partial_t a_m - \frac{\omega_m^2}{2}\sum_{l=1}^{N_\mathrm{f}}\delta\epsilon_l\left(a_{m-l}\mathbf{E}_{m-l}e^{i\theta_l} + a_{m+l}\mathbf{E}_{m+l}e^{-i\theta_l}\right)\right] = 0. \tag{S5}$$

Using the rotating wave approximation, taking a dot product with $\mathbf{E}_m^*(\mathbf{r})$ on both sides and integrating with the normalization $\int \mathbf{E}_m^*(\mathbf{r})\cdot\mathbf{E}_m(\mathbf{r})d\mathbf{r} = \omega_m$, we get

$$-i\partial_t a_m = \sum_{l=1}^{N_\mathrm{f}}\kappa_l a_{m-l} + \kappa_{-l}a_{m+l}, \tag{S6}$$

where

$$\kappa_{\pm l} = -\frac{1}{4n^2}e^{\mp i\theta_l}\int E_m^*(\mathbf{r})\cdot\delta\epsilon_l(\mathbf{r})E_{m\pm l}(\mathbf{r})d\mathbf{r} \tag{S7}$$

$$\simeq -\frac{1}{4n^2}\int_0^{2\pi}e^{\mp i(l\phi+\theta_l)}\delta\epsilon_l(\phi)d\phi\int r^2\sin\theta e^*(r,\theta)\delta\epsilon_l(r,\theta)e(r,\theta)drd\theta \tag{S8}$$

$$= -\frac{\alpha_l}{4n^2}\int_0^{2\pi}e^{\mp i(l\phi+\theta_l)}\delta\epsilon_l(\phi)d\phi, \tag{S9}$$

with $\alpha_l = \int r^2 \sin\theta e^*(r,\theta)\delta\epsilon_l(r,\theta)e(r,\theta)drd\theta$. Here we assume that all modes in the ring are of the form $E_m(\mathbf{r}) \simeq e(r,\theta)e^{-im\phi}$, i.e., the m-dependence of the modes under consideration of the unmodulated ring is predominantly from the azimuthal field profile.

Supplementary Note 2. Unitarity of $\mathcal{M}$

The transformation implemented by the ring-waveguide system from the input ports $\mathbf{s}^+$ to the output ports $\mathbf{s}^-$ is given by $\mathcal{M}$, where

$$\begin{aligned}\mathcal{M} &= \mathcal{I} + i\sqrt{2\Gamma}\left[\Delta\omega - i\Gamma - \mathcal{K}\right]^{-1}\sqrt{2\Gamma} \\ &= \mathcal{I} + 2i\left[\Delta\omega\Gamma^{-1} - i\mathcal{I} - \sqrt{\Gamma}^{-1}\mathcal{K}\sqrt{\Gamma}^{-1}\right]^{-1} &\text{(S10)} \\ &= \mathcal{I} + 2i\left[\Delta\omega\Gamma^{-1} - i\mathcal{I} - \tilde{\mathcal{K}}\right]^{-1} &\text{(S11)}\end{aligned}$$

where $\tilde{\mathcal{K}} = \sqrt{\Gamma}^{-1}\mathcal{K}\sqrt{\Gamma}^{-1}$. To show that $\mathcal{M}$ is unitary, we begin with writing the first term of Eq. (S11) as $\mathcal{I} = \left[\Delta\omega\Gamma^{-1} - i\mathcal{I} - \tilde{\mathcal{K}}\right]\left[\Delta\omega\Gamma^{-1} - i\mathcal{I} - \tilde{\mathcal{K}}\right]^{-1}$ to obtain

$$\mathcal{M} = \left[\Delta\omega\Gamma^{-1} + i\mathcal{I} - \tilde{\mathcal{K}}\right]\left[\Delta\omega\Gamma^{-1} - i\mathcal{I} - \tilde{\mathcal{K}}\right]^{-1} \tag{S12}$$

By writing the first term of Eq. (S11) as $\mathcal{I} = \left[\Delta\omega\Gamma^{-1} - i\mathcal{I} - \tilde{\mathcal{K}}\right]^{-1}\left[\Delta\omega\Gamma^{-1} - i\mathcal{I} - \tilde{\mathcal{K}}\right]$ we get another equivalent expression for $\mathcal{M}$ as

$$\mathcal{M} = \left[\Delta\omega\Gamma^{-1} - i\mathcal{I} - \tilde{\mathcal{K}}\right]^{-1}\left[\Delta\omega\Gamma^{-1} + i\mathcal{I} - \tilde{\mathcal{K}}\right] \tag{S13}$$

To verify that $\mathcal{M}$ is unitary, we use the expression for $\mathcal{M}$ from Eq. (S12) and for $\mathcal{M}^\dagger$ from Eq. (S13):

$$\begin{aligned}\mathcal{M}^\dagger\mathcal{M} &= \left[\Delta\omega\Gamma^{-1} - i\mathcal{I} - \tilde{\mathcal{K}}\right]\left[\Delta\omega\Gamma^{-1} + i\mathcal{I} - \tilde{\mathcal{K}}\right]^{-1}\left[\Delta\omega\Gamma^{-1} + i\mathcal{I} - \tilde{\mathcal{K}}\right]\left[\Delta\omega\Gamma^{-1} - i\mathcal{I} - \tilde{\mathcal{K}}\right]^{-1} \\ &= \left[\Delta\omega\Gamma^{-1} - i\mathcal{I} - \tilde{\mathcal{K}}\right]\mathcal{I}\left[\Delta\omega\Gamma^{-1} - i\mathcal{I} - \tilde{\mathcal{K}}\right]^{-1} \\ &= \mathcal{I}. &\text{(S14)}\end{aligned}$$

In this derivation, we have used the fact that $\mathcal{K}$ is Hermitian for a real refractive-index modulation $\delta\epsilon(\mathbf{r},t)$ and that Γ is Hermitian.

Supplementary Note 3. Truncation of modes in the synthetic frequency dimension

A ring resonator with a large circumference $L = 2\pi R$ supports a large number of resonant modes spaced approximately equally by the FSR. To achieve the high fidelities presented in this work, it is necessary to truncate the number of modes into which the input photons can couple so as to prevent the leakage of photons into undesired modes outside the $2N_{\mathrm{sb}}+1$-mode-wide band of interest. Here, we discuss in detail one method to achieve this truncation and numerically show its performance using a scattering matrix (S-matrix) analysis. For this purpose, consider a small auxiliary ring of length L_1 coupled to the main ring with a frequency-independent strength t_1, as shown in Supplementary Figure 1(a). We first discuss the unmodulated system. The S-matrices linking the fields at various points in the ring can be written as

$$\begin{pmatrix} a_3 \\ a_4 \end{pmatrix} = S_0 \begin{pmatrix} a_1 \\ a_2 \end{pmatrix} \tag{S15}$$

$$\begin{pmatrix} a_2 e^{-i\theta_0/2} \\ a_6 e^{-i\theta_1} \end{pmatrix} = S_1 \begin{pmatrix} a_5 e^{i\theta_0/2} \\ a_6 \end{pmatrix} \tag{S16}$$

where $\theta_0 = \beta(\omega)L + i\alpha L$ incorporates the effect of phase accumulation and amplitude attenuation as light propagates around the ring and $\theta_1 = \beta(\omega)L_1 + i\alpha L_1$ describes similar effects in the auxiliary ring. α is the propagation loss per

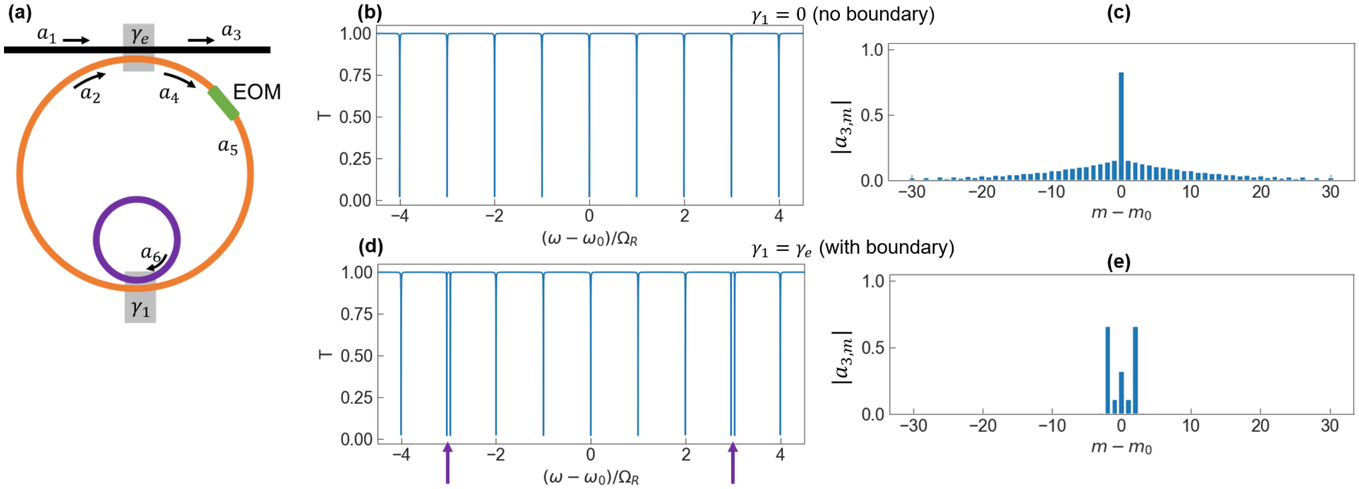

FIG. Supplementary Figure 1. Construction of boundaries in synthetic dimensions using auxiliary rings. (a) Schematic for S-matrix calculations, showing fields at various locations within the resonator. The ring-waveguide coupling is $\gamma_e = t_e^2/2T_R$, and the coupling between the main ring and the auxiliary ring is $\gamma_1 = t_1^2/2T_R$. The length of the main and auxiliary rings are L and L_1 respectively. (b) Steady-state transmission spectrum of the unmodulated ring without coupling to the auxiliary ring ($t_1 = 0$). Uniformly spaced modes separated by $\Omega_{\rm R}$ are seen. (c) Output spectrum for a modulated ring excited by a single input frequency at mode m_0 with no boundary $t_1 = 0$. The input spreads out into a large number of frequency modes since there is no boundary. (d) and (e) Same as (b) and (c) but with a boundary at modes $\omega_0 \pm 3\Omega_{\rm R}$, created by choosing $L_1 = L/6$ and $t_1 = t_e$. In (d), the ± 3 modes are split by the coupling to the auxiliary ring, as indicated by the purple arrows. In (e), we see that the output spectrum is localized to the five modes centered around m_0, since the modulation at the FSR is not able to couple across the boundary. 99.98% of the input power stays within the five modes defined by the boundaries. Note that in (b) and (d) we used an intrinsic loss $\gamma_i = \gamma_e$ for ease of visualization of resonant dips in transmission. In (c) and (e) we choose $\gamma_e \gg \gamma_i$.

unit length such that $\alpha L/T_R = \gamma_i$, where $T_R = 2\pi/\Omega_{\rm R}$ is the round-trip time. The matrices S_0 and S_1, which describe the directional couplers coupling the main ring to the external waveguide and to the auxiliary ring, respectively, have the form:

$$S_0 = \begin{pmatrix} \sqrt{1-t_e^2} & it_e \\ it_e & \sqrt{1-t_e^2} \end{pmatrix} \quad ; \quad S_1 = \begin{pmatrix} \sqrt{1-t_1^2} & it_1 \\ it_1 & \sqrt{1-t_1^2} \end{pmatrix} \tag{S17}$$

In the absence of modulation, $a_5 = a_4$. For a single frequency continuous wave excitation, we can solve Eqs. (S15) and (S16) by assuming $a_1 = 1$ and calculating a_3. The transmission spectrum T at the through-port of the ring is plotted in Supplementary Figure 1(b) and (d) without ($t_1 = 0$) and with ($t_1 = t_e$) the auxiliary ring, respectively. Without the auxiliary ring, the modes are equally spaced, showing resonances for $(\omega - \omega_0)/\Omega_{\rm R}$ being an integer. In the presence of an auxiliary ring with a length $L_1 = L/6$, every sixth mode is split into a doublet. Hence, a set of 5 modes are equally spaced, which can be coupled by the modulation at the FSR, but the split doublets at every sixth mode cannot be coupled by the modulation. This creates a one-mode boundary separating sets of 5 modes. We confirmed in our simulations that boundaries consisting of a larger number of modes can be formed by choosing a non-integer L_1/L and/or by using additional auxiliary rings. Note that for these calculations we assumed an intrinsic loss rate $\gamma_i = \gamma_e$ only to observe resonant dips in the transmission, but for high fidelity linear transformations, we set $\gamma_e \gg \gamma_i$, i.e. the ring is strongly over-coupled to the external waveguide. In this case, the transmission spectrum is near unity for both on and off resonance, but there are large on-resonance group delays.

In the presence of modulation, the fields a_5 and a_4 are coupled by the electro-optic modulator. In this case, each of the fields consists of multiple frequency components denoted as Floquet side bands, where the frequency of the Floquet sidebands are determined both by the input frequency $\omega_{\rm in}$ and the modulation frequency $\Omega_{\rm mod}$: $\omega'_m = \omega_{\rm in} + m\Omega_{\rm mod}$. Thus, the propagation phases $\theta_0(\omega)$ and $\theta_1(\omega)$ are dependent on the order of the Floquet sideband. The relation between the fields before and after the modulator in the S-matrix formalism can be obtained by exponentiating the

$\mathcal{K}$ matrix (see the discussion around Eq. (4) in the main text) from the coupled-mode theory:

$$S_K = e^{i\mathcal{K}T_R} \tag{S18}$$

$$a_{5,m} = \sum_n [S_K]_{m,n}\, a_{4,n} \tag{S19}$$

Using a large enough number of Floquet sidebands for calculations, the form of the matrix S_K for a single modulation frequency $\Omega_{\text{mod}} = \Omega_{\text{R}}$ (such that only κ_1 is nonzero) is:

$$S_K \approx \begin{pmatrix} \ddots & \vdots & \vdots & \vdots & \\ \cdots & J_0(\kappa_1 T_R) & J_1(\kappa_1 T_R) & J_2(\kappa_1 T_R) & \cdots \\ \cdots & J_{-1}(\kappa_1 T_R) & J_0(\kappa_1 T_R) & J_1(\kappa_1 T_R) & \cdots \\ \cdots & J_{-2}(\kappa_1 T_R) & J_{-1}(\kappa_1 T_R) & J_0(\kappa_1 T_R) & \cdots \\ & \vdots & \vdots & \vdots & \ddots \end{pmatrix} \tag{S20}$$

Using such a construction, we can calculate the steady state output frequency content for a certain input. In particular, we can check if the creation of boundaries in the synthetic dimension restricts the propagation of light to within the bounded set of modes without causing additional loss. We show this in Supplementary Figure 1(c) and (e) for the cases without a boundary and with a boundary respectively. In the absence of coupling to the auxiliary ring, the input at m_0 spreads out into a large number of modes (Supplementary Figure 1(c)), whereas in the presence of the boundary created by coupling to the auxiliary ring, 99.98% of the power stays localized within the 5 modes of interest (Supplementary Figure 1(e)). As in the unmodulated case, we checked that this behavior can be extended for multiple modulation tones by using non-integer values of L_1/L and/or by using additional auxiliary rings.

Supplementary Note 4. Performance under non-zero intrinsic loss

In this section, we consider the case where the intrinsic loss in the ring resonators is non-zero. In this scenario, the transformations implemented by the individual ring resonators of Fig. 1(b) are not unitary, and hence the overall transformation is not unitary. However, given a sufficient number of rings and modulation tones, it is still possible to use the system to implement a unitary matrix scaled by a constant. In Fig. Supplementary Figure 2 below, we repeat the optimization of permutation matrix of Fig. 4, but with $\gamma_m^i = 0.05\gamma_m^e$. With four modulation tones and one ring, the maximum achieved fidelity is $1 - 6.39 \times 10^{-2}$ with a scale factor of 0.9530, and the amplitudes of the obtained matrix are depicted in Supplementary Figure 2(b). Upon increasing the number of rings to four, the maximum fidelity improves to $1 - 3.85 \times 10^{-3}$ with a scale factor of 0.8691, and the amplitudes of the obtained matrix are depicted in Supplementary Figure 2(c). A full map of *one minus* the maximum fidelities as a function of the number of rings and modulation tones is shown in Supplementary Figure 2(d), indicating an expected decrease in fidelities in the presence of loss.

Supplementary Note 5. Performance with non-zero detuning

Here, we consider the case where the detuning $\Delta\omega$ is not the same for all rings in the system of Fig. 1(b). While the transformations implemented by the individual resonators remain unitary in the presence of such disorder, the fidelity of the overall system decreases since the excitation may not be on resonance for all rings. However, for reasonably small disorder, the overall system remains capable of approximating a variety of transformations. To emulate such disorder, we repeat the optimization of Fig. 5, but with the detuning $\Delta\omega$ of each ring chosen randomly between 0 and 10 (in units of γ) while the maximum modulation is limited to $|\kappa_l| \leq 5\gamma$. In Supplementary Figure 3(b) below, we show the element-wise phase of the obtained matrix with four modulation tones and one ring, achieving a fidelity of $1 - 3.84 \times 10^{-1}$. However, upon increasing the number of rings to four, the maximum fidelity improves substantially to $1 - 2.12 \times 10^{-6}$, as indicated in Supplementary Figure 3(c). A full map of *one minus* the maximum fidelities as a function of the number of rings and modulation tones is shown in Supplementary Figure 3(d). Despite the presence of random disorder that can exceed the modulation strength, the performance of the system remains remarkably robust, achieving near ideal fidelities for sufficiently large numbers of rings and modulation tones.

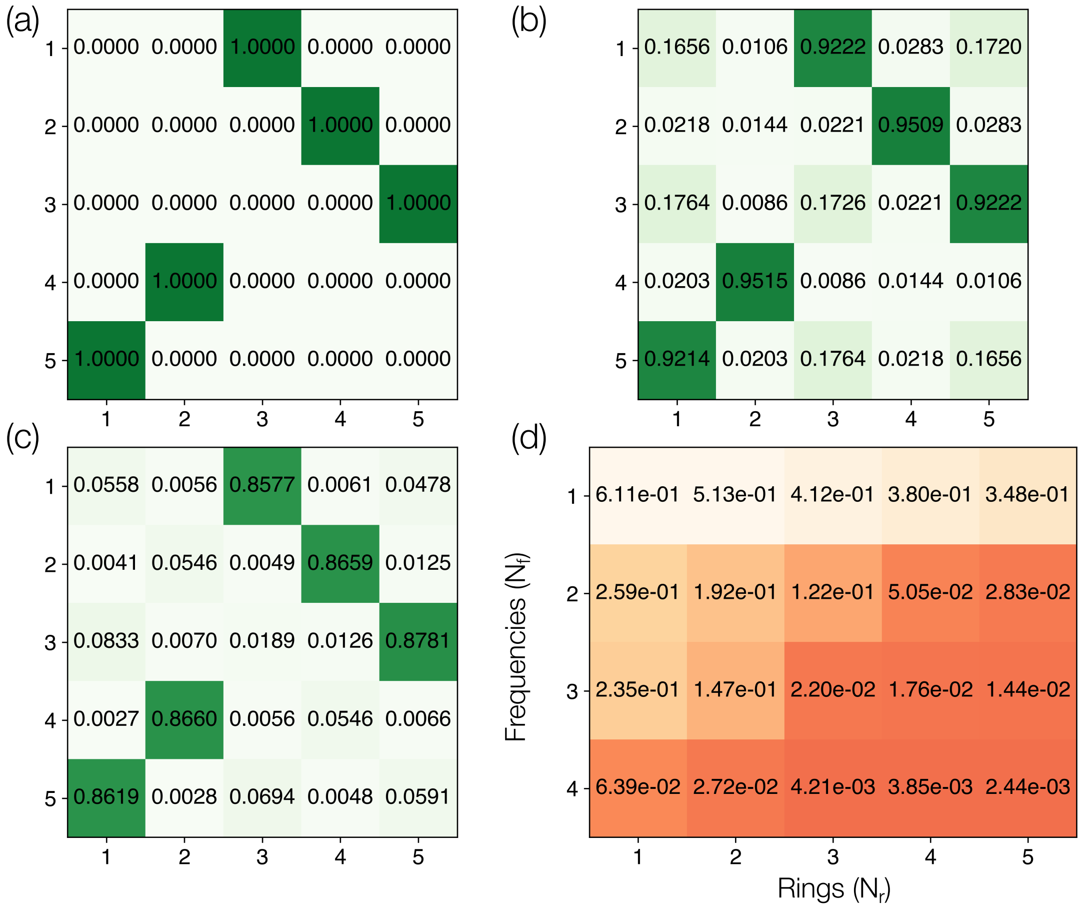

FIG. Supplementary Figure 2. (a) The 5×5 permutation matrix of Fig. 4 to be implemented by the ring-waveguide system under $\gamma_m^i = 0.05\gamma_m^e$. The amplitudes of the matrix elements are indicated along with a green colormap. (b) Element-wise amplitudes of the optimized result using four modulation tones ($N_\mathrm{f} = 4$) and (b) one ring ($N_\mathrm{r} = 1$), achieving a fidelity of $1 - 6.39 \times 10^{-2}$ with a scale factor of 0.9530, and (c) four rings ($N_\mathrm{r} = 4$), achieving a fidelity of $1 - 3.85 \times 10^{-3}$ with an overall scale factor of 0.8691. (d) *One minus* the maximum fidelities achieved by the inverse-design algorithm as a function of N_r and N_f. A value closer to zero indicates a better performance.

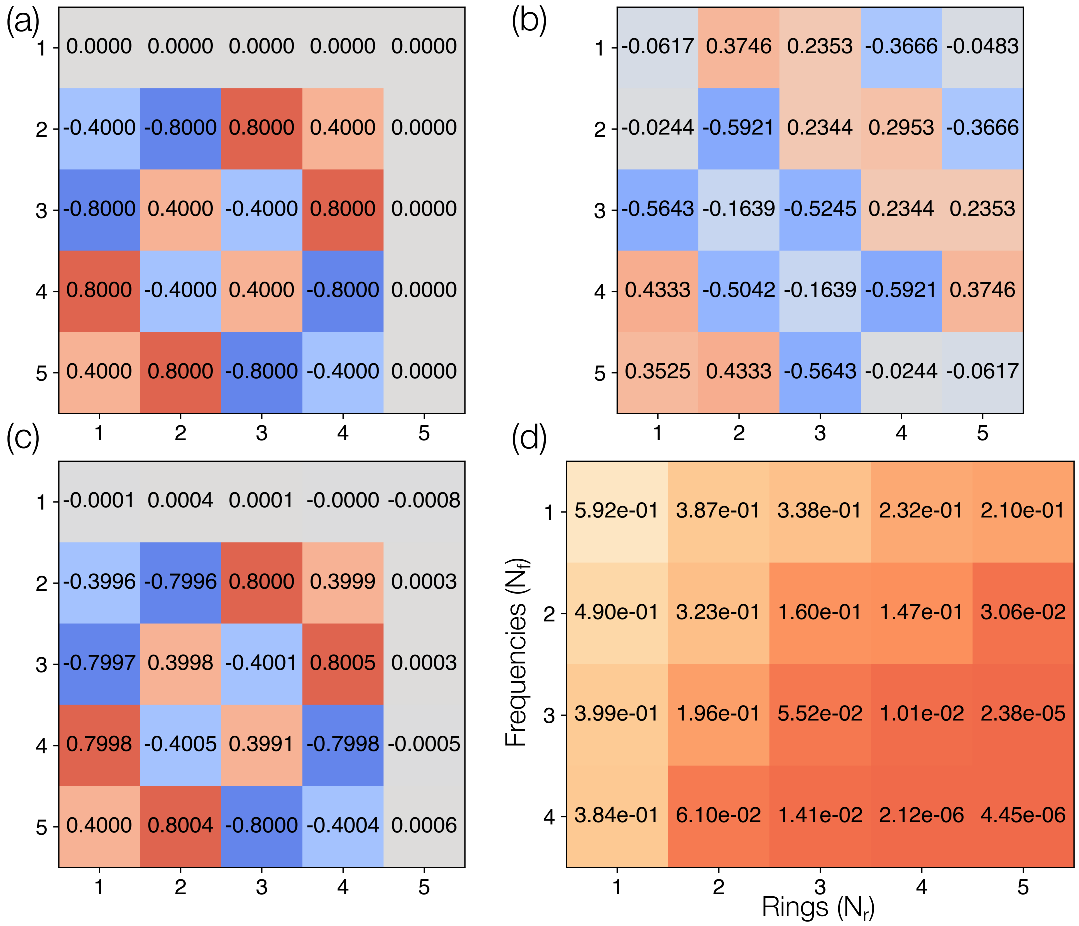

FIG. Supplementary Figure 3. (a) The 5×5 Vandermonde matrix of Fig. 5 to be implemented by the ring-waveguide system with detuning $\Delta\omega$ chosen randomly between 0 and 10γ for each ring. Element-wise phase achieved by the inverse-design algorithm for (b) $N_{\mathrm{r}} = 1$ and $N_{\mathrm{f}} = 4$, with a fidelity of 0.616 and (c) $N_{\mathrm{r}} = 4$ and $N_{\mathrm{f}} = 4$, with a fidelity of $1 - 2.12 \times 10^{-6}$. (d) *One minus* the maximum fidelities achieved by the inverse-design algorithm as a function of N_{r} and N_{f}. A value closer to zero indicates a better performance.

Supplementary Note 6. Parameters of the optimized systems

In this section, we list the parameters κ_l/γ found by the inverse-design algorithm that we use in Figs. 2-6.

**Fig. 2(b)**	Ring 1	Ring 2
κ_1/γ	$1.3508e^{0.2499\pi}$	$3.8269e^{0.2500\pi}$
κ_2/γ	$2.3574e^{i0.0\pi}$	$0.4878e^{i\pi}$

**Fig. 3**	Ring 1	Ring 2	Ring 3
κ_1/γ	$0.39e^{i0.3334\pi}$	$0.1469e^{i0.3331\pi}$	$0.6757e^{i0.3334\pi}$
κ_2/γ	$0.0001e^{i0.0\pi}$	$0.5152e^{i0.1666\pi}$	$0.0144e^{i0.1661\pi}$
κ_3/γ	$1.1031e^{i0.0\pi}$	$0.359e^{i0.0\pi}$	$1.0308e^{i0.0\pi}$

**Fig. 4(c)**	Ring 1	Ring 2	Ring 3	Ring 4
κ_1/γ	$0.1716e^{-i0.6197\pi}$	$0.1786e^{-i0.6772\pi}$	$0.2224e^{-i0.9549\pi}$	$0.3478e^{-i0.2951\pi}$
κ_2/γ	$0.1127e^{i0.3692\pi}$	$0.0820e^{-i0.4453\pi}$	$0.0503e^{i0.5313\pi}$	$0.2024e^{-i0.9075\pi}$
κ_3/γ	$0.0579e^{i0.2497\pi}$	$0.1852e^{i0.8489\pi}$	$0.1776e^{i0.8399\pi}$	$0.1010e^{i0.3586\pi}$
κ_4/γ	$0.2146e^{-i0.5535\pi}$	$0.0989e^{i0.0553\pi}$	$0.0057e^{i0.0915\pi}$	$0.0572e^{-i0.6616\pi}$

**Fig. 5(c)**	Ring 1	Ring 2	Ring 3	Ring 4
κ_1/γ	$0.5937e^{i0.2269\pi}$	$0.4273e^{i0.5323\pi}$	$0.8302e^{i0.3744\pi}$	$0.3750e^{i0.2093\pi}$
κ_2/γ	$0.5712e^{i0.3739\pi}$	$1.1299e^{i0\pi}$	$0.3310e^{i0\pi}$	$0.6499e^{i0.3417\pi}$
κ_3/γ	$0.8369e^{i0.2066\pi}$	$0.5694e^{-i0.6669\pi}$	$0.6474e^{-i0.6793\pi}$	$0.3388e^{i0.0561\pi}$
κ_4/γ	$1.4019e^{i0.3548\pi}$	$0.0756e^{i0.0\pi}$	$0.2493e^{-i0.776\pi}$	$1.0957e^{i0.0814\pi}$

**Fig. 6**	Ring 1	Ring 2	Ring 3	Ring 4
κ_1/γ	$0.6023e^{i0.6613\pi}$	$0.9449e^{i0.4425\pi}$	$0.1696e^{-i0.6438\pi}$	$1.208e^{i0.481\pi}$
κ_2/γ	$0.145e^{i0.9533\pi}$	$0.3338e^{-i0.8814\pi}$	$0.2973e^{i0.0\pi}$	$0.2441e^{-i0.2564\pi}$
κ_3/γ	$0.8391e^{-i0.3493\pi}$	$0.8065e^{-i0.954\pi}$	$0.4546e^{i0.4662\pi}$	$1.0546e^{i0.2438\pi}$
κ_4/γ	$0.4252e^{-i0.3689\pi}$	$1.7639e^{i0.1285\pi}$	$1.0045e^{i0.7818\pi}$	$0.6614e^{i0.6218\pi}$
